# Information-Seeking Patterns During the COVID-19 Pandemic Across the United States: Longitudinal Analysis of Google Trends Data

**DOI:** 10.2196/22933

**Published:** 2021-05-03

**Authors:** Tichakunda Mangono, Peter Smittenaar, Yael Caplan, Vincent S Huang, Staci Sutermaster, Hannah Kemp, Sema K Sgaier

**Affiliations:** 1 Surgo Ventures Washington, DC United States; 2 Department of Global Health & Population Harvard T.H. Chan School of Public Health Boston, MA United States; 3 Department of Global Health University of Washington Seattle, WA United States

**Keywords:** Google Trends, coronavirus, COVID-19, principal component analysis, information-seeking trends, information retrieval, trend, infodemiology, infoveillance, virus, public health, information seeking, online health information

## Abstract

**Background:**

The COVID-19 pandemic has impacted people’s lives at unprecedented speed and scale, including how they eat and work, what they are concerned about, how much they move, and how much they can earn. Traditional surveys in the area of public health can be expensive and time-consuming, and they can rapidly become outdated. The analysis of big data sets (such as electronic patient records and surveillance systems) is very complex. Google Trends is an alternative approach that has been used in the past to analyze health behaviors; however, most existing studies on COVID-19 using these data examine a single issue or a limited geographic area. This paper explores Google Trends as a proxy for what people are thinking, needing, and planning in real time across the United States.

**Objective:**

We aimed to use Google Trends to provide both insights into and potential indicators of important changes in information-seeking patterns during pandemics such as COVID-19. We asked four questions: (1) How has information seeking changed over time? (2) How does information seeking vary between regions and states? (3) Do states have particular and distinct patterns in information seeking? (4) Do search data correlate with—or precede—real-life events?

**Methods:**

We analyzed searches on 38 terms related to COVID-19, falling into six themes: social and travel; care seeking; government programs; health programs; news and influence; and outlook and concerns. We generated data sets at the national level (covering January 1, 2016, to April 15, 2020) and state level (covering January 1 to April 15, 2020). Methods used include trend analysis of US search data; geographic analyses of the differences in search popularity across US states from March 1 to April 15, 2020; and principal component analysis to extract search patterns across states.

**Results:**

The data showed high demand for information, corresponding with increasing searches for *coronavirus* linked to news sources regardless of the ideological leaning of the news source. Changes in information seeking often occurred well in advance of action by the federal government. The popularity of searches for unemployment claims predicted the actual spike in weekly claims. The increase in searches for information on COVID-19 care was paralleled by a decrease in searches related to other health behaviors, such as urgent care, doctor’s appointments, health insurance, Medicare, and Medicaid. Finally, concerns varied across the country; some search terms were more popular in some regions than in others.

**Conclusions:**

COVID-19 is unlikely to be the last pandemic faced by the United States. Our research holds important lessons for both state and federal governments in a fast-evolving situation that requires a finger on the pulse of public sentiment. We suggest strategic shifts for policy makers to improve the precision and effectiveness of non-pharmaceutical interventions and recommend the development of a real-time dashboard as a decision-making tool.

## Introduction

### Problem Statement

Over a period of a few months, the COVID-19 pandemic dramatically and rapidly changed the lives of most Americans. With the spread of SARS-CoV-2, people faced extreme uncertainty due to evolving information, calls for behavior change, and economic shocks. As US states move through different stages of the response and plan a path back to normalcy, it is important to develop ways to gather and measure insights into how Americans are responding to the challenges and uncertainties posed by the pandemic [1]. These insights will help policy makers know what to focus on as the United States continues to navigate the COVID-19 outbreak. In particular, it is important to understand the changes and patterns in the information seeking of Americans over the course of the pandemic, both as a window into key concerns as they emerge and as potential signals of actual behavior change.

Collecting data on population responses to a crisis poses a host of methodological challenges [2,3]. Surveys are a commonly used way to gather information on how people are reacting to a health crisis [4]. Several surveys have been deployed to measure Americans’ reactions to COVID-19, and they offer insights into the concerns and behaviors of many Americans [5,6]. However, traditional large-scale surveys are expensive and time-consuming; also, due to the rapidly changing nature of the pandemic, it is difficult to capture time-sensitive relevant data [4]. Further, surveys require a baseline understanding of the context of the participants before the events of interest. However, recent research [7] shows that many surveys administered during the COVID-19 pandemic either do not have a baseline or have an inaccurate baseline that relies on the participants’ limited ability to recall the answers to retrospective questions. Another option is to use health information systems, generating insights from existing large-scale health data such as electronic patient records and surveillance systems [8]. However, working with this type of data also has limitations: it requires multiple data sets that cannot easily be merged, it relies at times on outdated data, and it requires complex methodological approaches to analysis [9].

An alternative approach to data collection on pandemic response is to examine web-based information-seeking behavior through Google Trends, a source of data on trends in web-based information-seeking [10].

### Background

#### Google Trends and Health Phenomena

Google Trends data have been leveraged extensively in health behavior research by using trends in web-based search queries as proxies for changes in human behavior [11]. Released in 2006, these data became more influential when Ginsberg et al [12] showed a predictive approach that could “make it possible to use search queries to detect influenza epidemics in areas with a large population of web search users.” Since then, there have been suggestions to improve the predictive approaches after an overestimation of influenza metrics during the 2012-2013 season [13]. Outside of influenza predictions, Google Trends data have also been used to analyze a wide array of health behavior topics, such as the relationship between media coverage and information seeking around major infection events, the impact of local abortion policies on information seeking about abortion, the influence of influenza concerns on travel, and temporal patterns in interests in healthy behaviors such as dieting [14-17]. In 2014, Nuti et al [3] found that 60% of Google Trends research was focused on infectious diseases and general population behavior, which suggests that it may be a good tool to investigate COVID-19 trends.

Google Trends data are also useful for studying health information- and care-seeking patterns. Initial research using Google Trends data in the United States showed declining interest in imaging diagnostics [18] and surgeries [19] alongside a steady rise in interest in telehealth and telemedicine [20]. Other studies focused on COVID-19 and Google Trends data showed increases in general virus information seeking; however, to date, most studies have explored a singular issue over time or in a specific geographic region, without examining comprehensive search pattern correlations or geographical heterogeneity and their potential implications [21-23]. Recent research also shows “statistically significant correlations between Google Trends and COVID-19 data” [24]. Thus, Google Trends offers immense potential for generating temporally and geographically specific insights into the beliefs, behaviors, and actions of various communities in their response to the COVID-19 pandemic.

#### Health Information Seeking

Health information seeking is an important step in the care seeking journey, given that “one in three US adults use the internet to diagnose or learn about a health concern” [25]. Health information seeking has been extensively studied, with several studies identifying antecedents and reasons for individual health information-seeking behaviors. Factors that influence health information seeking include the subjective norms of a society or group of which the individual is a member [26], emotional responses and social context [27], and media-driven triggers [28]. Research on web-based health information seeking suggest certain reasons why individuals seek information on the web, including preparing for a doctor’s appointment [29] and anxiety about their health [30]. These studies are useful for some applications, such as improving the quality of information campaigns; however, they are general frameworks and are not disease specific.

In this work, we address information seeking from an infodemiology perspective, following seminal work by Eysenbach [30,31] that defines infodemiology as “the science of distribution and determinants of information in an electronic medium, specifically the Internet, or in a population, with the ultimate aim to inform public health and public policy”. We focus on the topics people search for (the demand side of infodemiology, in this case represented by Google Trends search queries) as opposed to what people write on the web (the supply side). Some infodemiology demand research focuses only on specific groups of people, such as the information-seeking behavior of clinical providers [32]. Our work instead examines the information seeking of the general population as measured by web-based search activity to capture wider public health implications. We also include a diverse set of socioeconomically related queries to address the multidimensional nature of the effect of COVID-19 and its intersection with nonpharmaceutical interventions. Analytically, we address three of four categories of analysis identified in a systematic review of Google Trends infodemiology by Mavragani et al [33]; we (1) measure the general web-based interest of several COVID-19– related themes, (2) detect variations and seasonality across geographic regions with comparisons to actual events, and (3) correlate search queries among them to understand the underlying patterns. The fourth category involves actual prediction and forecasting, which we do not address in this paper. In addition to specific public health and policy proposals, we emphasize the potential of an infoveillance [34] approach—using web-based information for public health surveillance—and propose a dashboard informed by this work.

### Research Questions

We created a curated list of search queries and used Google Trends to analyze changes in information seeking related to the COVID-19 pandemic over time and across geographic areas in the United States. We answered four broad questions regarding information seeking around COVID-19:

First, how is information seeking changing over time? Specifically, what is the relationship between the COVID-19 outbreak and internet searches related to health care seeking, government support programs, media sources of different ideologies, planning around social activities, travel, and food, and new COVID-19–specific behaviors and concerns? We would expect certain types of searches (eg, health care–related inquiries, government safety net programs, web-based food delivery) to become more popular, while others would be expected to fall in popularity (eg, nearby bars, travel plans). These trends provide clear contextual information on the changing interests and concerns of Americans as the pandemic progresses, and we can also compare the timing of these changes with real-world actions and events such as policy announcements.

Second, what geographic variation exists in information-seeking behavior? Specifically, how does the popularity of search terms differ across states and regions? Given the immense heterogeneity across the United States, both culturally and in terms of COVID-19 caseload and response, we would expect states to differ in the most-sought information. Observed geographic differences in information seeking add granularity to the information-seeking context and provide an opportunity to develop hypotheses around why these differences exist.

Third, do states have particular and distinct patterns in information seeking? Specifically, which searches correlate with each other at the state level? Using machine learning methods, specifically unsupervised learning, we can construct different typologies of geographic areas so that those whose search trends provoke concern for public health can be better targeted with information and other interventions.

Fourth, do Google Trends data correlate with and potentially precede real-life events? If Google Trends data can be “predictive” of real-life events (eg, unemployment rates), it provides further validation for this method as a window into the behaviors of Americans during the pandemic.

## Methods

Google Trends is a free web-based source of data on trends in web-based information seeking across geographies (country, region, city, and designated market area/metropolitan area where this exists) and over time (since 2004). In 2019, a comprehensive methodological framework was published to standardize approaches to Google Trends research [35]. This work follows that framework, which provides specific criteria for selecting the keywords, geographical regions, and time period for analysis. We also combine several keywords that represent a similar topic into a single query.

### Keywords and Search Queries

Google Trends accepts a single word or a phrase, and several of these can be combined into a single Trends query by joining with a “+”, which functions like an “OR.” This study used 38 queries of up to 5 words/phrases each (Table 1). We generated and curated search terms and evaluated the popularity/relevance and data quality of each query in representing different aspects of the response to and impact of COVID-19–related search activity on Google Trends. The list of search terms was derived from the most commonly searched terms associated with COVID-19 on Google Trends [10] and was refined to be more specific as recommended by Tran et al [36].

Each query was then categorized into one of six emergent themes: care seeking, government programs, health programs, news and influence, outlook and concerns, and social and travel (Table 1). These themes were developed to comprehensively capture the effects of and responses to the pandemic: care seeking and health programs capture how people look for care; news and influence captures the sources of information; and outlook and concerns and social and travel capture the economic and nonpharmaceutical preventive intentions. For the news and influence theme, we selected media outlets that are representative of both sides of the ideological spectrum based on a Pew Research survey conducted in 2014 that measured the ideological leanings (left or right) of the audiences of different news media sources [37]. All search data were extracted on April 15, 2020.

**Table 1:** Final list of search queries grouped into emergent themes (38 queries with 1-5 combined terms).

Category/ theme	Queries
Care seeking	*coronavirus symptoms* *coronavirus testing near me* + *coronavirus testing center near me* + *coronavirus test* *doctor appointment* *coronavirus afford doctor* + *coronavirus uninsured* + *coronavirus medical bill* *Coronavirus can i see a doctor* + *coronavirus can i get a test* + *coronavirus are tests available* *doctor open* + *doctor office open* *urgent care near me*
Government programs	*disability benefits* + *apply benefits* + *food stamps* + *wic* *government aid* *recession* + *stock market crash* + *economic downfall* + *bear market* *small business loans* *stimulus check* *paycheck protection program*
Health programs	*health insurance* *health insurance* + *medicare* + *medicaid* *medicaid* *medicare*
News and influence	*chinese virus* *coronavirus cnn* + *coronavirus msnbc* + *coronavirus nbc news* + *coronavirus cbs news* *coronavirus hoax* + *coronavirus fake news* *coronavirus infowars* + *coronavirus breitbart* + *coronavirus glenn beck* + *coronavirus the blaze* *coronavirus washington post* + *coronavirus new york times* + *coronavirus npr* *coronavirus fox news* + *coronavirus drudge report*
Outlook and concerns	*can’t pay rent* + *how pay rent* + *behind on rent* + *can't pay mortgage* *hoarding* + *hoard* *how can i stop coronavirus* *how to make coronavirus mask* *how to stockpile* + *buy in bulk* + *bulk order* *sick days* + *sharing sick days* + *no sick days* + *sick leave* + *paid time off* *social distancing* *sold out* + *stock out* + *stockout* + *stockpil* *unemployment benefits* + *unemployment application* + *file unemployment* + *apply unemployment* + *layoffs*
Social and travel	*bar closed* + *restaurant closed* *bar near me* + *restaurant reservation* + *local happy hour* + *ladies’ night* *bar* + *restaurant* + *happy hour* + *pub* + *house party* + *party ideas* *cheap flights* + *travel destinations* + *flight deals* + *vacation deals* *food delivery* + *grocery deliveries* + *takeout* + *curbside* + *online food order* *house party* + *party ideas*

### Data Collection

Data from Google Trends [10] were extracted through an open-source third party Python application programming interface, PyTrends. Google provides the relative search values (RSVs) for each query on a scale from 0 to 100, representing a normalized value.

Three primary data sets were developed from Google Trends: one at the national level and the other two at state level, capturing all 50 states and the District of Columbia.

The national data set contained weekly RSVs for the United States for each of 38 queries for the 224 weeks between January 1, 2016, and April 15, 2020. This enabled us to conduct trend analysis for each query independently, tracking its relative popularity in the entire United States compared with its most popular week over these 224 weeks. For each query, the week when the query was most popular (as a percentage of all queries in that week) was scored as 100 by Google Trends, and all other weeks were scored relative to this week.

One of the state-level data sets contained weekly RSVs for each query for each state for the 16 weeks between January 1 and April 15, 2020, while the other contained aggregated RSVs for each query for each state over the whole period of March 1 to April 15, 2020. For each of these data sets, RSVs for a specific query were always expressed as search popularity relative to other weeks, other states, or both, but never relative to other search queries (ie, our approach factored out absolute differences in popularity between queries).

Finally, two additional, secondary data sets were referenced, comparing Google Trends against new monthly Medicaid applications and weekly initial unemployment claims, from the public Medicaid web-based database (June 2017 to January 2020) [38] and US Department of Labor data (January 2016 to April 2020) [39], respectively.

### Analysis

Geographically, states were grouped by federal region and division based on US census data [40]. We used Python for data processing and Tableau for exploratory data analysis and visualization [41]. We used the packages of Python and R (R Foundation for Statistical Computing) to perform pairwise correlations (Pearson, pairwise complete) and unsupervised learning by principal component analysis (PCA), respectively [42]. We conducted pairwise correlations to visualize and quantify associations between the individual queries at the state level; we then applied dimensionality reduction through PCA to reveal groups of queries that are often searched together and to identify top states with search patterns of interest, either supporting or undermining the fight against the COVID-19 epidemic. Missing values were removed for correlation and PCA analysis (ie, we retained all 51 geographies and removed any queries that had missing values for any states). Nine queries had missing values: *coronavirus infowars + coronavirus breitbart + coronavirus glenn beck + coronavirus the blaze, how can I stop coronavirus, coronavirus can I see a doctor + coronavirus can I get a test + coronavirus are tests available, coronavirus afford doctor + coronavirus uninsured + coronavirus medical bill, bar closed + restaurant closed, government aid, doctor appointment, doctor open + doctor office open,* and *can't pay rent + how pay rent + behind on rent + can't pay mortgage*. This reduced the number of queries for analysis from 38 to 29.

For pairwise correlations, we filtered for any correlations of low to very high (positive or negative) correlation (ie, >0.3 or <–0.3) according to the guideline proposed by Mukaka [43]. We then focused only on the most prominent (moderate and high) scales. For PCA, only loadings with loading scores higher than 0.2 or lower than –0.2 were used to explain each component (PCA loadings take values from 0 to 1).

To calculate the increase in search popularity between January and March 2020, we took the mean RSV for March 2020 and divided this by the mean RSV for January 2020 and expressed this ratio as a percentage change. We chose January and March to capture the largest shifts in RSV based on the progression of the SARS-CoV-2 virus. Data for the extra 2 weeks in April were used to monitor which trends persisted or dissipated after large shifts in March.

Finally, to compare Google Trends to real-life phenomena, we visualized data on actual weekly initial unemployment claims and new applications for Medicaid, then quantified the correlations between each phenomenon and the corresponding Google Trends query.

## Results

### Magnitude of Shifts in Information Seeking

National trends show shifts in information seeking related to health and lifestyle activities.

**Figure 1:**
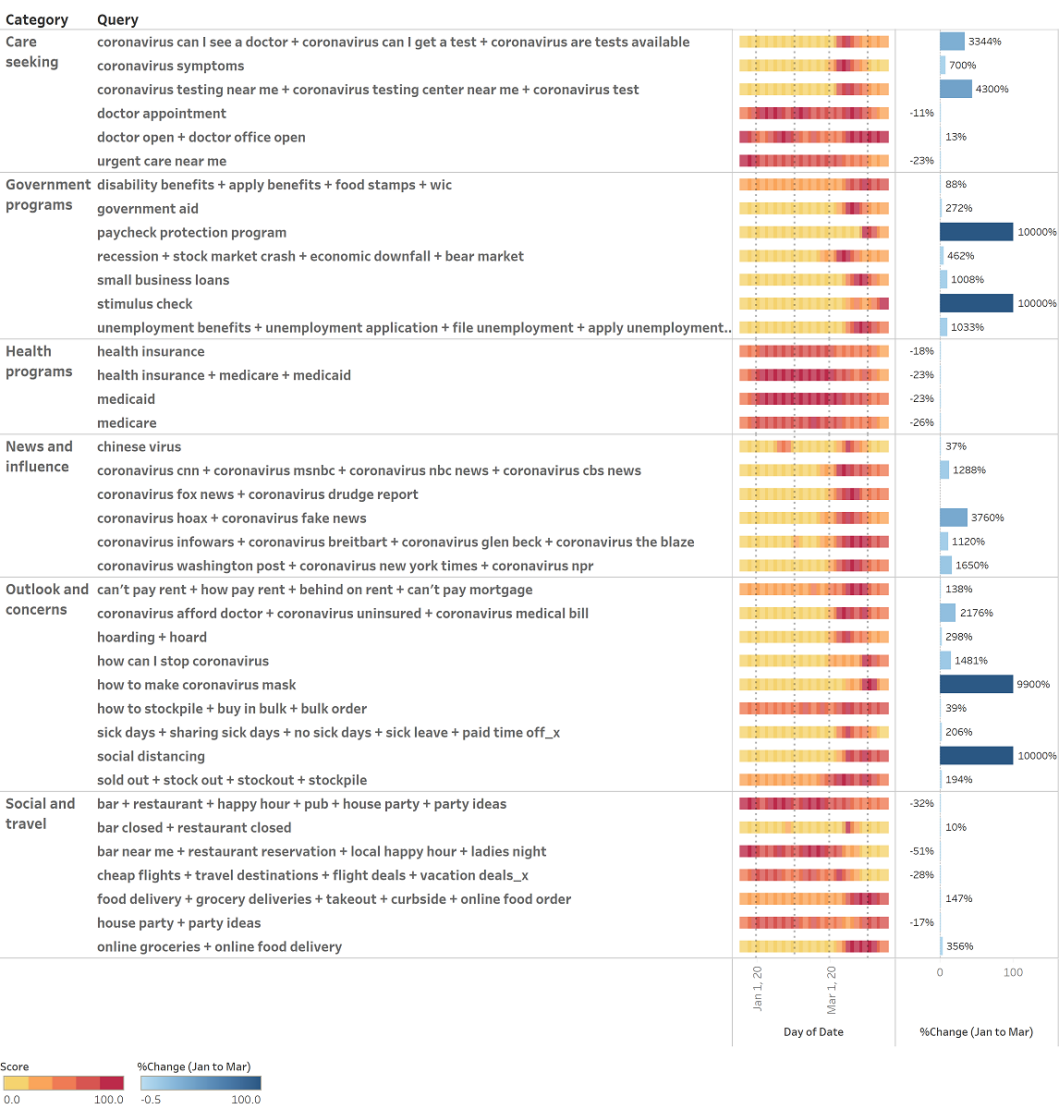
National search pattern for each query in every theme by relative search value for 16 weeks between January 1 and April 15, 2020. The right panel shows the change in monthly average relative search value between January and March, capped at 10,000% for queries with extremely high changes.

Analysis of all queries showed substantial shifts in RSVs across all thematic categories (Figure 1) predominantly in March 2020, with trends persisting or stabilizing in early April.

For the care seeking theme, the RSVs of COVID-19–related queries increased substantially (*coronavirus symptoms* and *testing centers*) while the RSVs of general health-seeking queries (*urgent care* and *doctor appointments*) declined in late March/early April, suggesting a nuanced story of care seeking (Figure 1). This trend was matched by a decline in the RSVs for all queries in the health programs theme (*health insurance*, *medicaid*, and *medicare*) by 18%, 23%, and 26%, respectively. Comparison with the same time window in preceding years confirmed that this finding was an anomaly against historical seasonality, where health program searches peak and drop in November and December, not in March and April.

News and influence searches related to COVID-19 saw notable hikes in RSV for both left-leaning and right-leaning media. RSV for far-right/alt-right media outlets (see the *Methods* section for definitions based on the Pew Research survey) also increased. Simultaneously, RSV for *coronavirus fake news* and *coronavirus hoaxes* surged 38-fold (Figure 1).

For the outlook and concerns theme, RSVs for new behavioral concepts gained immense popularity: *social distancing* and *how to make masks* spiked by 100-fold and peaked in March, as did time-sensitive concerns such as *hoarding* and *can’t pay rent*. In contrast, searches for *stock outs/sold out* and *coronavirus medical bill/affordability* remained at high levels into early April, hinting at potential differences in long-term versus short-term concerns (Figure 1).

Within the social and travel theme, search trends were aligned with the new norm of social distancing and also signaled potential drops in business for the travel and service industries. The RSVs for *online groceries* + *food delivery* and *food delivery* + *grocery deliveries* + *takeout* + *curbside* + *online food order* tripled and doubled, respectively, while the RSVs for *party ideas*, *nearby bars and restaurants*, and *cheap flights/travel* all dropped by between 17% and 51%. This shift is noteworthy for *nearby bars and restaurants*, as it is a reversal of an upward trajectory that had recently peaked in December 2019 (Figure 1).

For the government programs theme, its relative popularity increased for new COVID-19–specific packages (*stimulus check* and *small business loans*) as well for unemployment benefits, which is a more mature program. Notably, the RSVs for *disability/food stamps* and *government aid* had the lowest increases in RSVs among government programs. However, relative interest in programs focused on individuals—*stimulus check* and *unemployment application*—maintained momentum through April 15 (Figure 1).

### Timing of Shifts in Information Seeking

**Figure 2:**
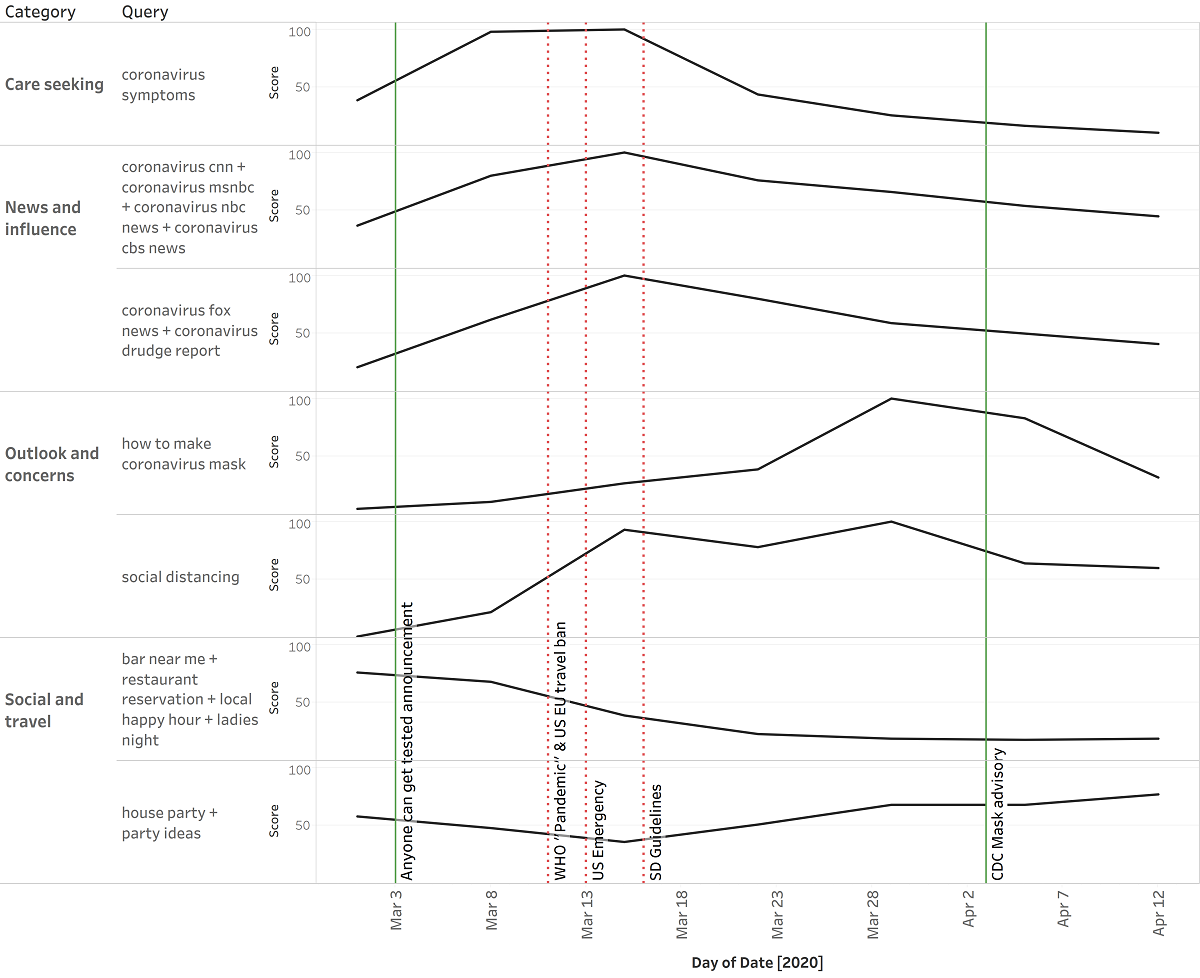
National trends for Google Trends nonpharmaceutical intervention–related queries compared to actual government and public health nonpharmaceutical interventions at the US federal level between March 1 and April 15, 2020. CDC: US Centers for Disease Control and Prevention; EU: European Union; SD: social distancing; WHO: World Health Organization.

Shifts in information seeking occurred earlier than federal government policy action. We would have expected the directives from the federal government, which formulates national policies, to be issued early and to precede changes in search patterns and behaviors. However, the Google Trends data show that the relative popularity of queries related to non-pharmaceutical interventions (NPIs) was already shifting substantially, and in some cases peaking, several days ahead of major federal government policies and NPI action (Figure 2). For example, RSVs for *social distancing* and *bar/restaurant nearby* were quickly increasing and decreasing, respectively, by March 8—3 days before the World Health Organization declared COVID-19 a pandemic, 5 days before the US federal government’s national emergency declaration, and 8 days before the government released official social distancing guidelines. Furthermore, *how to make a coronavirus mask* was already relatively popular (RSV >50) by March 23, exactly 12 days before the US Centers for Disease Control and Prevention issued an advisory promoting the use of masks for the general public.

On the other hand, the RSV trends for news on COVID-19 and COVID-19 symptoms were in sync, matching each other regardless of the ideological leaning of the news source. This suggests that searches for disease characteristics were highly correlated with searches for news coverage of the disease.

### Correlation Between Information Seeking and Unemployment and Medicaid Claims

**Figure 3:**
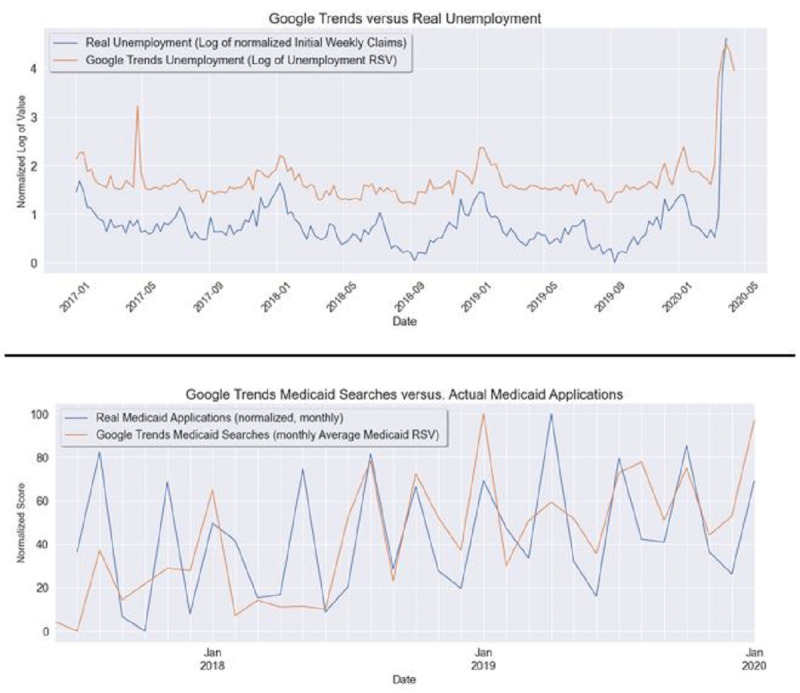
National-level multivariate trends for new weekly unemployment (January 2016 to April 2020) [39] and new monthly Medicaid applications (July 2017 to January 2020) [38]. Top: Google searches for unemployment applications and actual initial weekly unemployment claims normalized from 0-100 over time (with seasonal adjustment). Bottom: Monthly average Google searches for Medicaid and actual new applications for Medicaid normalized from 0-100 (lagged by 1 month). RSV: relative search value.

Google Trends were correlated with unemployment claims and Medicaid applications. We found high correlations between specific Google Trends queries and corresponding phenomena in the real world both before the epidemic (for unemployment and Medicaid) and during the epidemic (for unemployment). Two examples were selected: initial weekly unemployment claims and new monthly Medicaid applications. Figure 3 shows the comparative results with corresponding Google Trends queries over time. 

There was a very high positive correlation of 0.96 between the previous week’s Google Trends RSV for unemployment applications and the actual weekly initial unemployment claims normalized to 0-100 (seasonally adjusted as recommended by the Bureau of Labor Statistics to eliminate seasonal spikes and enable easy detection of nonseasonal anomalies such as COVID-19–related spikes). For Medicaid, there was a moderate positive correlation of 0.55 between the average monthly Google Trends RSV for Medicaid, lagged one month, and the number of new applications for Medicaid each month.

However, we noted that this approach was only modestly successful for more complex cases in which the link between search popularity and actual behavior is more difficult to imagine. A notable example is stock market prices (Dow Jones Industrial Average) and the Google Trends query for *recession/stock market crash*, which showed no correlation.

### Differences in Search Patterns Across States and Regions

Differences in search patterns suggest varied responses to COVID-19 across states and regions. Nationally, the largest jumps in RSV were concentrated between March 1 and April 15; therefore, we focused on this window to investigate state-by-state differences in information seeking. Here, we compared the relative popularity of a search query across states in a single time period rather than across time.

**Figure 4:**
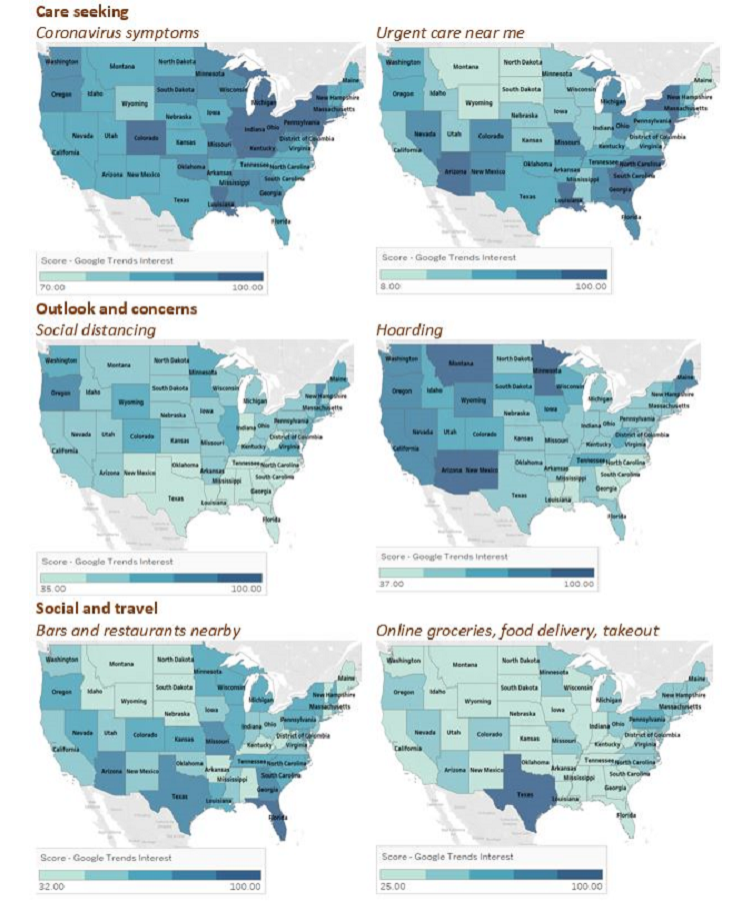
Geographical variations across states from March 1 to April 15, 2020, for the care seeking, outlook and concerns, and social and travel themes. The figure shows the two queries that were most representative of each category.

The findings showed similar levels of popularity of searches for *urgent care near me* in the South and Northeast but a difference in the relative popularity of searches for health programs (*health insurance*, *Medicare*, and *Medicaid*). Care seeking searches were most popular in the South (Louisiana, Georgia, and North Carolina); the Northeast (New York and New Jersey); and in Indiana, Illinois, and Arizona during this specific window (Figure 4). Although New York and New Jersey were already COVID-19 hotpots, this period also coincided with sharp increases in the number of cases for Louisiana, Georgia, and North Carolina.

RSVs for *social distancing* were generally high in the Northeast and West but generally low in the South, while searches for *hoarding* were relatively more popular in Alaska, New Mexico, Minnesota, Arizona, and Montana than in other states during this period.

**Figure 5:**
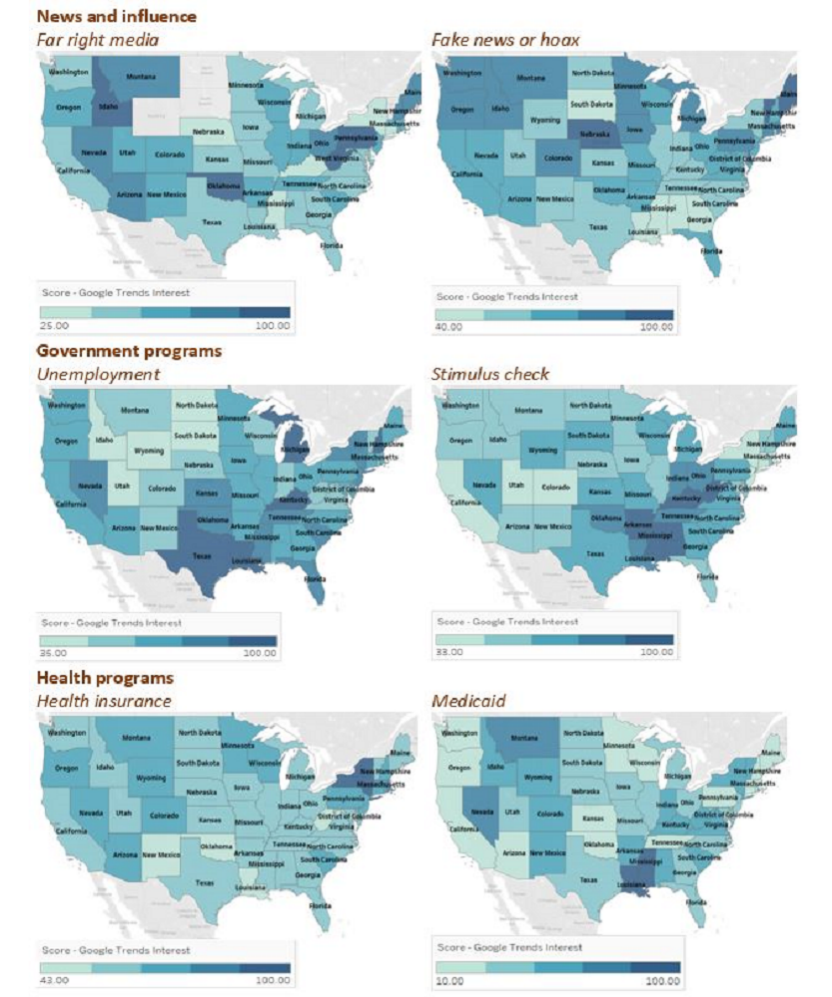
Geographical variations across states from March 1 to April 15, 2020, for the news and influence, government programs, and health programs themes. The figure shows the two queries that were most representative of each category.

Regarding the news and influence theme, searches for *far-right/alt-right* and *coronavirus* were most popular in West Virginia, Oklahoma, Idaho, and Pennsylvania, while *fake news coronavirus* searches were most popular in DC, Vermont, Alaska, Maine, and Nebraska (Figure 5). Additionally, *stimulus check* had particularly high RSVs in Southern states (Figure 5). Although *health insurance* had a high RSV in the Northeast (New York, Massachusetts, and Vermont), *Medicaid* had higher RSVs in Southern states, including Louisiana and Mississippi (Figure 5).

### Summarizing Differences in Search Patterns Using Correlation and PCA

Much variability in information seeking between states can be explained by a few components.

Although the thematic categories in the above analysis give insights on the relative popularity of queries over time and across geographic regions, we also conducted pairwise correlation and PCA analyses to understand how the queries were correlated. First, correlation analysis showed that states with high RSVs for *coronavirus symptoms* also tended to have high RSVs for *urgent care* and *test centers*, suggesting a relationship between awareness of the disease and potential intention to seek care. Second, the correlation showed that states with high RSVs for *social distancing* also tended to have high RSVs for *coronavirus* and all types of news media regardless of ideological leaning, *recession/stock market crash*, and *sick days/leave*, while tending to have low RSVs for safety net programs such as *medicare* and *stimulus check*. This finding suggests that news sources and economic factors play a role in the levels of interest in, awareness of, and potential adoption of social distancing behavior.

**Figure 6:**
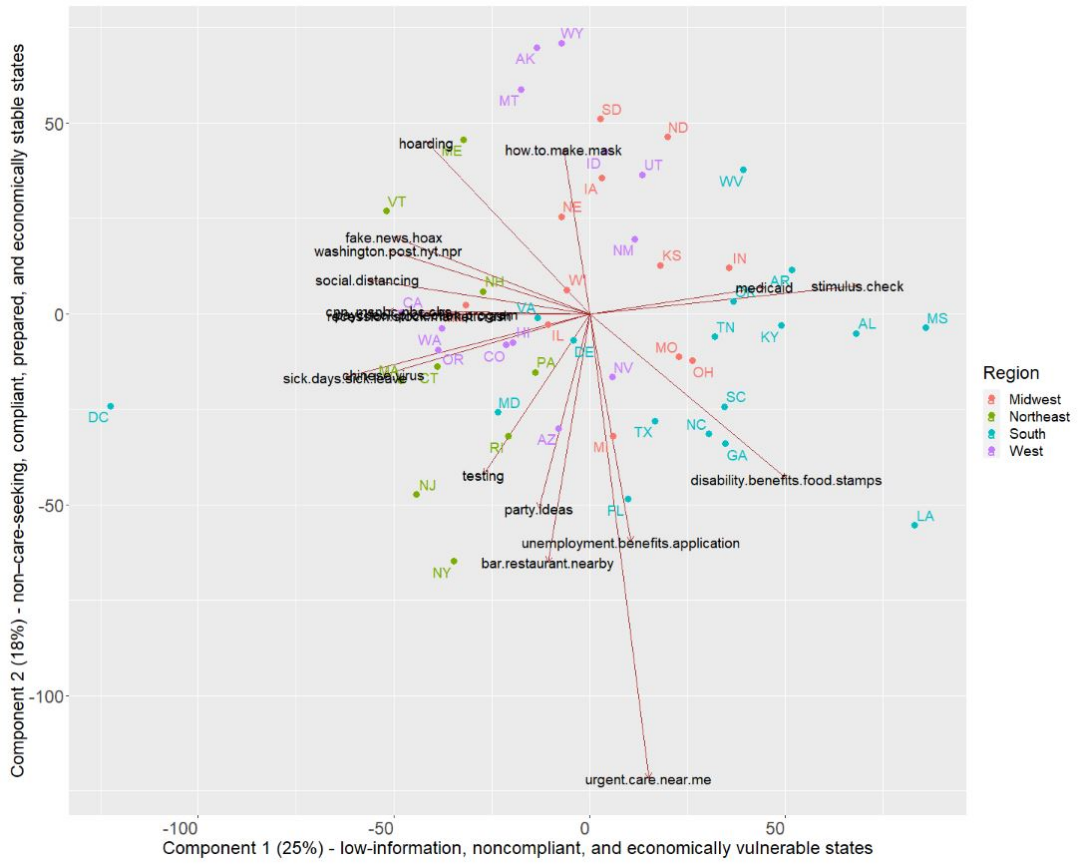
Scatterplot of state principal component analysis loadings and scores for the first two components, with the top queries shown as arrow vectors. Each arrow represents the relative weight of each query, and the direction indicates the points to the states that most exhibit this search pattern. The arrow direction measures correlation; arrows in the same direction are highly positively correlated, while divergent arrows in opposite directions are highly negatively correlated. Component 1 (x-axis) and Component 2 (y-axis) explain 25% and 18% of the variation (differences or patterns) in the data, respectively.

The PCA results are shown in Figure 6, with all 38 queries summarized as the top two components that explain 43% of the variation (differences or patterns) in the data—25% from Component 1 and 18% from Component 2. The two components were assigned labels based on the search patterns they showed.

PCA revealed search patterns related to economic vulnerability and searching for information from news and media sources. It also highlighted how state search patterns were related to other concepts, such as compliance with social distancing or related policies such as mask wearing; preparation for emergencies (hoarding); and care-seeking concepts, such as searching for urgent care.

Specifically, Component 1 explained 25% of the variation in the data (Figure 6). This component represented potentially low-information, noncompliant, and economically vulnerable states*.* These terms are defined as follows: *low-information*: low association with searches for any news source, whether real or fake; *noncompliant*: low association with searches for *social distancing*; and *economically vulnerable*: high association with searches for *disability/food stamps* and *stimulus check*. The states with the highest scores (ie, those that exhibited this search pattern most strongly) were Mississippi, Louisiana, Alabama, Arkansas, and Kentucky. Additional clusters of states within Component 1 also had high scores for *urgent care nearby* and *unemployment application* (Florida and Michigan) as well as *disability/food stamps* (Georgia, North Carolina, and South Carolina).

Component 2 explained 18% of the variation in the data (Figure 6). This represented potentially non–care-seeking, compliant, prepared, and economically stable states*.* These terms are defined as follows: *non–care-seeking*: low association with searches for *urgent care nearby*; *compliant*: low association with searches of *nearby bars/restaurants*; *prepared*: high association with searches for *how to make a mask* and *hoarding*; and *economically stable*: low association with searches for *unemployment* and *disability/food stamps*. The states exhibiting this trend were Wyoming, Alaska, Montana, North Dakota, and South Dakota.

## Discussion

### Principal Findings

We studied the demand side of the infodemiology [31] of COVID-19 using a curated set of queries from Google Trends grouped into emergent themes. Our findings show substantial changes in COVID-19 information seeking at the national level, particularly in March 2020, suggesting a hyperawareness and desire for information about both COVID-19 and its corresponding novel behavioral concepts, namely social distancing and mask wearing. This is in line with other studies showing spikes in information seeking during disease outbreaks [44]. The trends also mirrored the rapid changes in the way people eat, travel, and socialize. The high demand for information corresponded with increasing searches for news sources and coronavirus, regardless of ideological leaning, including searches for *coronavirus fake news* + *coronavirus hoax*. It is not conclusive whether this indicates curiosity or an earnest belief in the existence of “fake news.” Still, this finding underscores the critical and timely role that news sources play in providing information during a pandemic and why this information must be correct and trustworthy. This complements other Google Trends studies [45,46] that showed that information seeking and collective attention toward COVID-19 can, at times, be driven by media coverage more than epidemic trends.

Effective communication during health crises is critical and, if not done well, leads to “public confusion and misunderstanding” with negative public health consequences, as happened with COVID-19 in the United States [47,48]. Our study found that changes in information seeking often occurred well in advance of action by the federal government. Tracking search patterns for social distancing or mask wearing could show public health authorities what interventions people are aware of and might accept, as well as the best time to start talking about them, especially in rapidly evolving situations. Previous studies showed that attention paid to COVID-19–related information spiked sharply but quickly saturated despite high media coverage [44] as the pandemic continued to spread. Therefore, the right interventions, if poorly timed, could be too early to be acceptable, or, as occurred in the case of the COVID-19 pandemic, too late to be optimally effective.

The decrease in popularity of searches related to urgent care, doctors’ appointments, and health insurance/Medicare/Medicaid is consistent with the decline in health seeking that occurs during pandemics: prospective patients for other diseases have higher risk perception of hospital-based transmission of COVID-19, which reduces their health-seeking behavior [49]. This was especially true in the United States, where hospitals prioritized COVID-19 and relegated other services [50]. This finding can be an early-warning indicator of reduced health-seeking behavior, especially for vulnerable communities with underlying health risks. It can be used to prioritize and target these communities with messages about the continued importance of health care for conditions and symptoms not related to COVID-19.

State-level differences in search patterns confirmed the trajectory of the pandemic and informed our potential hypotheses about the regional and structural drivers of these differences. For example, in New York and New Jersey, two states where the pandemic quickly accelerated and where, coincidentally, health insurance marketplaces are state-run rather than federally run, people were performing searches related to urgent care and health insurance [51]. Residents of southern states tended to search for several potentially worrying factors in the fight against COVID-19, including searches related to urgent care and Medicaid and searches for stimulus checks, indicating the need for a financial safety net. The search for *hoarding* was popular in states with either large land areas (and less dense populations) or especially extreme weather conditions—Alaska, New Mexico, Minnesota, Arizona, and Montana. This may reflect heightened expectations of scarcity of the populations of these states during a pandemic, as these factors likely make these states hard to reach in the case of disrupted supply chains. These hypotheses need to be validated; however, they provide a starting point for anticipating the trajectory of the next pandemic or national emergency.

Using correlation and PCA, we identified economic and financial factors, access to information, and interest in social distancing as key variables in describing states' information-seeking patterns on COVID-19. Using this premise, Mississippi, Louisiana, Alabama, Arkansas, and Kentucky were identified as low-information, noncompliant, and economically vulnerable states during the time window of the analysis (March and April 2020). Thus, the states that are most vulnerable economically or in terms of the social safety net are also the least informed and show the least search-related interest in social distancing, a key intervention to prevent COVID-19. This insight can be used to increase awareness of social distancing and its benefits while targeting and prioritizing resources to support these states, including increased testing, health system capacity support, and economic relief measures.

To capture the evolving information and insights available through Google Trends, we propose a real-time dashboard to track trends, geographical variations, and patterns of interest regarding the epidemic. This proposal is in line with evidence that Google Trends data are statistically significantly correlated with COVID-19 data [24]. The queries used in this study can be used as a starting point or baseline, with additional features added if needed. As the epidemic continues and potentially gives way to a second wave, this dashboard will follow how specific queries change over time and which states demonstrate concerning search patterns using the pairwise correlation and PCA approaches. Depending on the search patterns identified, policy makers can then design or improve interventions as well as allocate resources to target states.

### Limitations

We made some assumptions and choices that may result in limitations depending on the use case. First, the selection of keywords and queries was an iterative process because it is difficult to know a priori what search terms best capture a desired concept. Analytically, we used weekly RSVs as opposed to daily RSVs, resulting in some loss of granularity. We also used the state as our main unit of analysis and not the city or designated market area level because the COVID-19 response was organized at the state level and lower levels had more missing values. However, at state level, there were a few missing values that were included in the trend and geographical analyses but removed for the pairwise correlation and PCA analyses.

While valuable insights can be derived, a major limitation of Google Trends is that it will always be a measure of search patterns and not the actual corresponding behaviors; therefore, inference and prediction must always come with this caveat. Finally, this analysis is limited by specific assumptions of Google Trends, which include pulling the data from only a sample and not the whole database of searches and providing it in the form of RSV instead of absolute search volumes per geographic region. This requires caution when analyzing results from low-volume queries or geographic regions and when making interpretations and conclusions from analyses.

### Conclusion

Our work provides insights into, and potential indicators of, important changes in information-seeking patterns during pandemics such as COVID-19 that can be used to inform public health and public policy. High demand for information corresponding with increasing search popularity for COVID-19 from news sources highlights the importance of public health authorities working with media to ensure that information is correct. Decreases in search popularity for health seeking–related searches can be an early warning indicator for policy makers to target areas with serious underlying health risks with messages about the importance of continuing to seek care for other ailments, even during a pandemic. The emergence of economic and financial factors, access to information, or interest in social distancing as important variables suggests potential interventions to increase awareness of social distancing and its benefits, while targeting and prioritizing resources to support states, including increased testing, health system capacity support, and economic relief measures.

We demonstrated that PCA can explain the variation across several queries and multiple geographies. This work underscores the importance of tracking a well-curated set of queries, customized for the topic, and combined to capture all angles of the problem to improve public health in real time.

## Abbreviations

NPI: nonpharmaceutical intervention
PCA: principal component analysis
RSV: relative search value
